# Metagenomic Study of Fungal Microbial Communities in Two PDO Somontano Vineyards (Huesca, Spain): Effects of Age, Plant Genotype, and Initial Phytosanitary Status on the Priming and Selection of their Associated Microorganisms

**DOI:** 10.3390/plants12122251

**Published:** 2023-06-08

**Authors:** Natalia Langa-Lomba, Jerome Grimplet, Eva Sánchez-Hernández, Pablo Martín-Ramos, José Casanova-Gascón, Carmen Julián-Lagunas, Vicente González-García

**Affiliations:** 1 Instituto Universitario de Investigación en Ciencias Ambientales de Aragón (IUCA), EPS, University of Zaragoza, Carretera de Cuarte s/n, 22071 Huesca, Spain; natalialangalomba@gmail.com; 2 Departamento de Sistemas Agrícolas, Forestales y Medio Ambiente, Centro de Investigación y Tecnología Agroalimentaria de Aragón (CITA), Avda. Montañana 930, 50059 Zaragoza, Spain; cjulian@aragon.es; 3 Instituto Agroalimentario de Aragón-IA2 (Universidad de Zaragoza-CITA), 50059 Zaragoza, Spain; jgrimplet@cita-aragon.es; 4 Departamento de Ciencia Vegetal, Centro de Investigación y Tecnología Agroalimentaria de Aragón (CITA), Avda. Montañana 930, 50059 Zaragoza, Spain; 5 Department of Agricultural and Forestry Engineering, ETSIIAA, Universidad de Valladolid, Avenida de Madrid 44, 34004 Palencia, Spain; pmr@uva.es; 6 Instituto Agroalimentario de Aragón-IA2 (Universidad de Zaragoza-CITA), EPS, University of Zaragoza, Carretera de Cuarte s/n, 22071 Huesca, Spain; jcasan@unizar.es

**Keywords:** NGS sequencing, endophytic mycobiota, GTDs, fungal diversity

## Abstract

The study of microbial communities associated with different plants of agronomic interest has allowed, in recent years, to answer a number of questions related to the role and influence of certain microbes in key aspects of their autoecology, such as improving the adaptability of the plant host to different abiotic or biotic stresses. In this study, we present the results of the characterization, through both high-throughput sequencing and classical microbiological methods, of the fungal microbial communities associated with grapevine plants in two vineyards of different ages and plant genotypes located in the same biogeographical unit. The study is configured as an approximation to the empirical demonstration of the concept of “microbial priming” by analyzing the alpha- and beta-diversity present in plants from two plots subjected to the same bioclimatic regime to detect differences in the structure and taxonomic composition of the populations. The results were compared with the inventories of fungal diversity obtained by culture-dependent methods to establish, where appropriate, correlations between both microbial communities. Metagenomic data showed a differential enrichment of the microbial communities in the two vineyards studied, including the populations of plant pathogens. This is tentatively explained due to factors such as the different time of exposure to microbial infection, different plant genotype, and different starting phytosanitary situation. Thus, results suggest that each plant genotype recruits differential fungal communities and presents different profiles of associated potential microbial antagonists or communities of pathogenic species.

## 1. Introduction

Grapevine crops are frequently threatened by a range of phytosanitary problems. Among these, grapevine trunk diseases (GTDs) have stood out, particularly in the last 2–3 decades [1], due to various factors. These include the gradual withdrawal of chemical substances that allow their control, the increase in newly planted areas (resulting in more production of starting plant material), and the intensity of current crop management (such as pruning, planting densities, irrigation, fertilization, driving, etc.) [2,3].

GTDs are characterized by great complexity in terms of their etiology. Syndromes attributable to GTDs are typically multifaceted, with the existence of symptoms common to several diseases, several etiological agents acting simultaneously or sequentially, or the continuous appearance of new pathologies and species associated with them [1]. The etiological agents responsible for these types of pathologies are usually ubiquitous and polyphagous fungal pathogens, often living in alternative crops and refuge plant species or colonizing the inner parts of the woody tissues of the grapevine as endophytes or latent parasites (part of the usual endophytic microbiota of the plant) [4]. Their attack and symptoms appear randomly in time and space, generally as a result of imbalance phenomena in the plant’s immune system, and are typically due to the generation of management stresses [5].

Until barely a decade ago, approaches to characterize and understand the role played by microbial communities associated with different agroecosystems, such as grapevines [6], employed classical microbiological methodologies dependent on the use of a limited number of axenic culture media available for the isolation of microorganisms. This procedure has limited the characterization only to cultivable microbial diversity, which represents a small fraction of the total number of microbes associated with the soil, rhizospheres, or host tissues of plants that they colonize [7,8]. Nowadays, with the generalization of different massive sequencing techniques, high-throughput sequencing analyses have made it possible to reveal aspects such as the total microbial diversity existing in agricultural soil, the relationships between communities, the biological potential of the agroecosystem, or the key microorganisms associated with the different types of phenotypic response observed against exposure and infection by different pathogens [9]. In this last approach, the plant genotype is considered as a part of a whole that must also integrate all the microbial communities that live connectedly with it. The role of these communities in the biology and behavior of the host has only begun to be elucidated in the last 10–15 years. In this way, the next-generation sequencing (NGS) analyses of microorganisms associated with grapevine crops have suggested that these microbial communities and numerous specific pathogens of each plant host can evolve and be different depending not only on the different agroecological and management conditions but also on the genetic profile of each variety, largely due to the processes of interaction and selection (“priming”) of certain microbial diversity promoted by the host plant, which would have been selected to confer adaptive advantages against all kinds of stresses, including diseases [10,11,12].

The importance and the role played by the different communities of microorganisms associated with agroecosystems have recently been focused on with a phytopathological viewpoint. This offers a new paradigm where plants are considered not only as an individual genotype but also as a larger genetic entity that includes their associated microorganisms (microgenome), which has given rise to the new concept of “holobiont” [13]. Under this perspective, a holobiont must be considered as a genetic set comprised of the individual plant and its symbionts, as well as other associated microbes, acting as a single unit of biological organization. The microbiome is compartmentalized into the rhizosphere, endosphere, phyllosphere, carposphere, and other non-specific endophytic microbiota, according to the different plant tissues colonized by microorganisms [14]. In addition, aspects such as the metabolism and morphology of a given plant species and its microbiota are closely related to each other to maintain the ecological fitness of the holobiont [15].

The use of high-throughput sequencing techniques has allowed for the characterization of microbial communities associated with the grapevine crop and has led to several studies focused on its ecological functionalities, management, breeding, and disease control [16]. Some of these studies have focused on the relationships between the plant microbiome and its phytosanitary status [6] or the influence of different vineyard management practices on the composition of microbial communities [17]. Other studies compared the microbiomes associated with crops of different ages [18], plant genotypes, phenological stages [19], or plant tissues [20,21]. Metagenomic studies in microbial diversity have also identified potential biocontrol methods of GTDs using microbial antagonists of fungal or bacterial origin [22,23]. Finally, several works revealed that plant health is overall a direct consequence of the composition and balance of its associated microbial communities [6]. Therefore, the ecological fitness of the grapevine holobiont, including its behavior and resilience against a series of biotic and abiotic stresses, is modulated by the composition of its microbiome, which could serve as a biological marker [24]. Furthermore, metagenomic studies analyzing different compartments of the soil–plant interface in grapevine plants have identified several niches that have the potential for colonization and infection by certain soil-borne fungi associated with GTDs [20]. This finding demonstrates the preeminent role of the different compartments of the rhizosphere and its surrounding soil in the dispersal and spread of certain plant pathogens.

The objective of this study was, in the first step, to characterize the fungal microbial communities associated with two conventional vineyards of different ages that belong to the same biogeographical unit. This was accomplished by comparing both culture-dependent (direct isolation on synthetic media and characterization of endophytic fungal strains) and high-throughput sequencing methods. The aim was to detect dissimilarities between the composition of fungal communities and the factors that influenced them. Both culture-dependent techniques and high-throughput sequencing methods were utilized to compare the existing diversity between the two agroecosystems of different ages and plant genotypes. This approach allowed for the experimental examination of the concept of microbial priming by the plant host, revealing and explaining differences in the structure and composition of the microbial communities. These differences could be attributed to various factors, including the functional activity of the different genotypes present in each vineyard, the duration of exposure to infection and microbial colonization, the origin of the plant material, and the initial phytosanitary situation. Collectively, these factors influence and shape the selection of microorganisms observed in each of the analyzed plots. The experimental verification of these hypotheses highlights the significance and relevance of the holobiont concept and its functionality. It underscores how plant genotypes interact directly with the microbial genome to selectively include or exclude microorganisms that contribute, positively or negatively, to the maintenance of overall ecological fitness.

## 2. Results

### 2.1. Isolation of Endophytic Fungi from Grapevine Plants

Fungal communities present in the sampled grapevine individuals were characterized using culture-dependent microbiological methods. In the two vineyards sampled, a total of 240 endophytic fungal isolates were obtained (136 belonging to the plants surveyed in the “Clau” plot and 104 obtained from the “Almendros” vineyard). After taxonomic characterization of the different fungal operational taxonomic units (OTUs) employing both classical and molecular methods, it was found that the “Clau” vineyard yielded a higher number of endophytic isolates than the “Almendros” vineyard, with 44 and 35 fungal strains identified, respectively (Tables S1 and S2). Despite these differences in the number of characterized isolates, both surveyed plots had similar proportions between pathogenic grapevine-associated taxa and saprophytic ubiquitous endophytic species found. This ratio was found to be about 70/30% in the “Clau” vineyard and 60/40% in the “Almendros” vineyard. However, the composition of fungal genera and species exhibited some interesting differences between them. Putting aside some common grapevine pathogens associated with adult plants, which were isolated from both vineyards and represented by fungi responsible for the so-called ‘Botryosphaeria dieback’ (i.e., *Neofusicoccum parvum* (Pennycook and Samuels) Crous, Slippers and A.J.L. Phillips, and *Diplodia seriata* De Not.) and ‘eutypiose’ (*Eutypa lata* (Pers.) Tul. and C. Tul.), there were remarkable differences in other GTD-related pathogens. In this sense, some species and genera were isolated exclusively in one of the two vineyards analyzed. Thus, a conventional microbiological survey of the “Clau” plot revealed the presence of some species associated with the so-called ‘Petri disease’, such as *Phaeoacremonium aleophilum* W. Gams, Crous and M.J. Wingf. or *Phaeomoniella chlamydospora* (W. Gams, Crous, M. J. Wingf. and L. Mugnai) Crous and W. Gams, which were not isolated in the sampling of plants in the “Almendros” vineyard. Interestingly, analyses of this latter plot revealed the presence of several isolates of *Cylindrocarpon macrodidymum* Schroers, Halleen and Crous, a member of the so-called ‘black foot’ disease, which is mostly associated with young grapevine plants, together with the presence of one of the etiological agents responsible for grapevine excoriose, *Phomopsis viticola* Sacc.) Sacc.

Concerning other types of plant pathogenic endophytes obtained in both plots, it is worth noting the presence of other polyphagous species such as *Discostroma fuscellum* (Berk. and Broome) Huhndorf and *Didymella glomerata* (Corda) Qian Chen and L. Cai (the latter also present in the “Almendros” vineyard) in the “Clau” plot or *Didymosphaeria variabile* (Riccioni, Damm, Verkley and Crous) Ariyawansa and K.D. Hyde and *Phoma conidiogena* Schnegg in the “Almendros” plot. In addition, other ubiquitous endophytes, either saprophytic or facultative pathogens belonging to genera such as *Fusarium*, *Gibberella*, *Aspergillus*, *Rhizopus*, etc., were also repeatedly isolated. Finally, the presence of isolates belonging to genera commonly reported in the literature as microbial antagonists with the potential for use as biological control agents (BCAs) (genera *Trichoderma* and *Aureobasidium*) was also observed in both plots.

### 2.2. NGS Analyses of Fungal Communities

**Figure 1:**
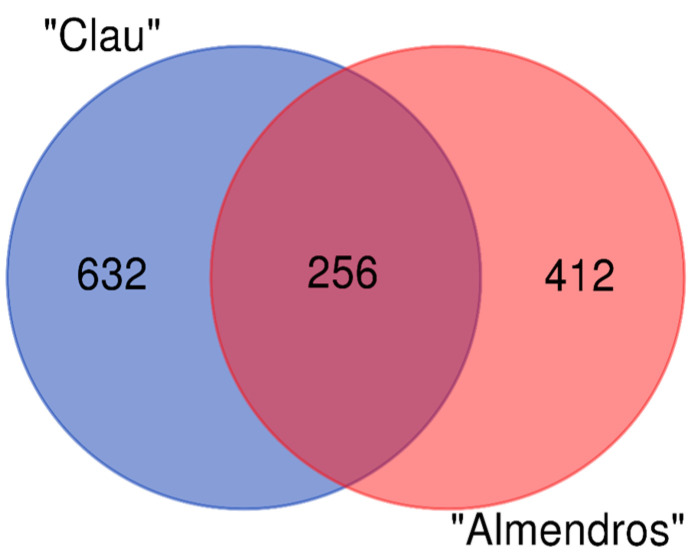
Venn Diagram showing the number of unique OTUs identified in each of the vineyards analyzed and those shared by both.

Together with analyzing the fungal diversity in grapevine plants using classical microbiological methods, the microbial diversity of internal wood samples coming from the same plant stands in two vineyards of different ages and phytosanitary status of the PDO ‘Somontano’ was characterized using high-throughput sequencing techniques. A metagenomic taxonomical analysis based on sequences of the ITS2 fragment from the ribosomal ITS (Internal Transcribed Spacer) region was conducted, resulting in ITS sequences from a total of 1300 OTUs (Table S3), with 635 binomials representing 318 taxa. Of the total OTUs identified, 632 were exclusive to the “Clau” vineyard and 412 were only found in the younger plot (the “Almendros” vineyard). Therefore, 25% more taxa were identified in the older plot (23 vs. 8 years). In addition, 256 OTUs (approximately 20% of the total) were common to both plots (Figure 1).

**Figure 2:**
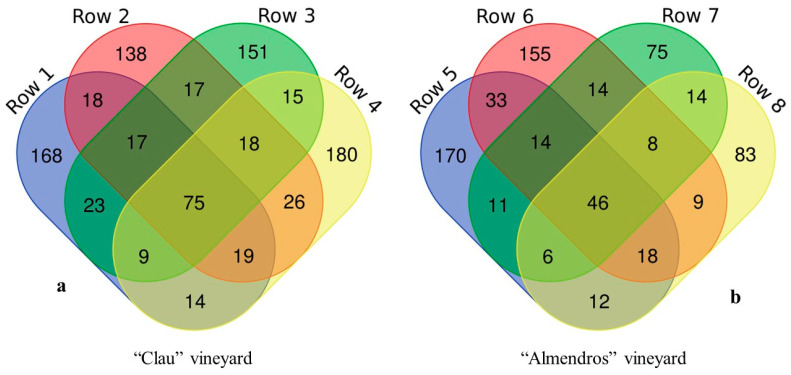
Venn Diagram showing the different common and unique OTUs present in the different rows sampled per plot for the two vineyards analyzed: (**a**) the “Clau” vineyard, rows 1–4; (**b**) the “Almendros” vineyard, rows 5–8.

Regarding the common and exclusive taxa present in each of the analyzed plots (Figure 2), it was observed that the number of taxa common to all plants analyzed was higher (75 OTUs) in the older “Clau” plot compared to the younger “Almendros” vineyard, where only 46 common OTUs were detected, representing almost 40% fewer.

When comparing all the sampled plant stands, including the two experimental plots, a total of 30 OTUs that were common to all the surveyed rows were identified (Table 1). Sixteen of these OTUs were grapevine-associated taxa, with varying degrees of specificity, which mainly consisted of specific pathogens (e.g., *Phaeomoniella chlamydospora*, *Cladosporium cladosporioides* (Fresen.) G.A. de Vries, *Seimatosporium vitis* Y.P. Xiao, Camporesi and K.D. Hyde, and *Neosetophoma lunariae* Crous and R.K. Schumach), ubiquitous or cosmopolitan endophytic species (e.g., *Aureobasidium pullulans* (de Bary) G. Arnaud, *Epicoccum nigrum* Link, *Alternaria alternata* (Fr.) Keissl., Beih., etc.), or miscellaneous taxa (e.g., *Cystofilobasidium macerans* Samp., *Vishniacozyma victoriae* (M.J. Montes, Belloch, Galiana, M.D. Garca, C. Andrs, S. Ferrer, and Torr.-Rodr. and J. Guinea) X.Z. Liu, F.Y. Bai, M. Groenew. and Boekhout, *Filobasidium stepposum* (Golubev and J.P. Samp.) Xin Zhan Liu, F.Y. Bai, M. Groenew. and Boekhout, etc.), which were somehow associated with the crop and cited in previous studies of a similar nature [11,25,26,27]. Taking into account the range of frequency values obtained and the fact that some of the OTUs common to all sampled rows were taxa usually associated with fruits (grapes), leaves, or even flowers (without being specifically associated with the interior of the woody tissues of the plant), and that others represented cosmopolitan fungal endophytes frequent in all types of plant hosts, the analysis of the grapevine’s inner wood microbiome did not allow the recognition of a true “core” population or essential microbiome in the two investigated vineyards. Among the OTUs common to all the samplings specifically associated with grapevine, only *Phaeomoniella chlamydospora*, a well-known specific pathogen associated with the vascular rot of grapevine wood, showed reading frequencies above 10,000. In this sense, the most frequent common taxa associated with vines (although with significantly lower values than those reported for *P. chlamydospora*) were essentially pathogenic species such as *Cladosporium cladosporioides*, responsible for grape rot [28], or *Seimatosporium vitis*, associated with GTD pathologies [29]. In summary, the essential microbial core inhabiting the inner wood and characterized for the two investigated vineyards seemed to be dominated, according to the present analysis, by pathogenic species present to a lesser or greater degree and frequency of appearance, followed by generalist or ubiquitous endophytic taxa, together with a series of species associated with the host, although not specifically with the type of tissue or organ analyzed.

As expected, and in accordance with what has been reported in most inventories on endophytic fungal diversity [6], the majority presence of identified OTUs belonging to Div. Ascomycota (871) was detected in both plots, revealing a lower number (354) of OTUs from Basidiomycota. In addition, Div. Mortierellomycota was barely represented in the analysis, with the detection of only three OTUs, and no representative of the Oomycetes group (Kingdom Chromista) was found. Finally, a small number of OTUs (11) representing taxa from Div. Chytridiomycota were also identified.

The analysis of the metagenomic results revealed that almost 80% of the total identified taxa consisted of rare and/or infrequent taxa (with relative frequencies below 2%). The microbiota of the younger plot turned out to be less diverse, dominated by relatively few species that were highly represented in the plants sampled.

Regarding the pathogenic component of the fungal microbial communities analyzed, the results showed that *Phaeomoniella chlamydospora*, one of the etiological agents of the so-called Petri disease, was the most represented species in the metagenomic analysis, with a slightly higher frequency in the older “Clau” vineyard (16% of the reads) compared to the younger “Almendros” vineyard (13% of the reads). However, other members of the Petri complex, such as taxa from genera *Phaeoacremonium* (e.g., *P. aleophilum* and *P. minimum* (Tul. and C. Tul.) Gramaje, L. Mostert and Crous) and *Cadophora* (e.g., *C. luteo-olivacea* (J.F.H. Beyma) T.C. Harr. and McNew and *C. malorum* (Kidd and Beaumont) W. Gams), were scarcely represented. The genus *Eutypa* and other Xylariales were the second most frequent group of species associated with GTDs in both vineyards. Another group of taxa associated with ‘Botryosphaeria dieback’, including genera *Neofusicoccum*, *Dothiorella*, and *Diplodia*, were more abundant in the older plants (“Clau” vineyard). However, despite the age of the oldest vineyard analyzed (23 years for the “Clau” plot), the analysis did not reveal the presence of OTUs representing any of the lignicolous basidiomycete taxa commonly associated with GTD syndromes such as grapevine esca in our latitudes (e.g., *Fomitiporia mediterranea* M. Fisch., *Inonotus hispidus* (Bull.) P. Karst., or *Stereum hirsutum* (Willd.) Pers.). Instead, the analysis characterized the presence of sequences belonging to some other genera of *Hymenochaetaceae* such as *Fuscoporia* (*F. ferruginosa* (Schrad.) Murrill), *Fomitiporella*, or *Phellinus* (*P. rhamni* (Bondartseva) H. Jahn), which have been related to esca symptoms in vine-producing areas such as Chile or South Africa [30] or cited in the metagenomic fungal inventories of grapevine [9].

#### 2.2.1. Alpha-Diversity of Fungal Microbiome

**Figure 3:**
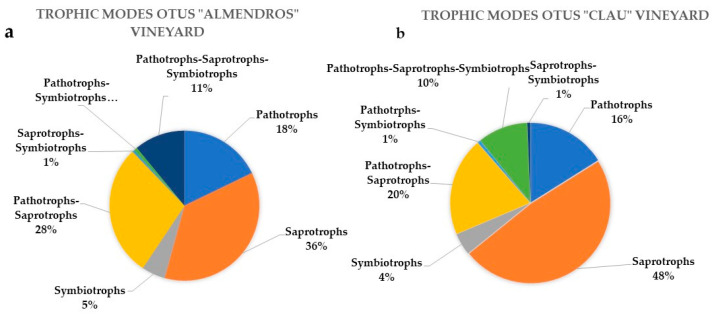
Circle charts showing the type and percentage of different trophic modes associated with the OTUs characterized for the two vineyards analyzed: (**a**) “Almendros” vineyard; (**b**) “Clau” vineyard.

The metagenomic analyses conducted on the two vineyards showed that the percentages of species associated with different lifestyles, guilds, and nutritional modes (Table S4) were equivalent regardless of the age of the experimental fields (Figure 3). The percentage of OTUs representing pathogenic lifestyles was similar in both plots, representing 16% and 18% in “Almendros” and “Clau”, respectively. The percentage of saprophytic taxa was slightly higher in the older plot, with 48% of the OTUs compared to 36% in the youngest vineyard. As expected, and depending on the type of sample and plant organ surveyed, the number of symbiont taxa was low in both plots, representing 5% and 4% in “Almendros” and “Clau”, respectively, and was dominated by lichenizing species, which were probably associated with DNA contamination coming from grapevine barks during sampling. When considering OTUs with a wider range of trophic modes, including combinations between types of nutrition (according to the available literature), the percentages between vineyards were also similar.

**Figure 4:**
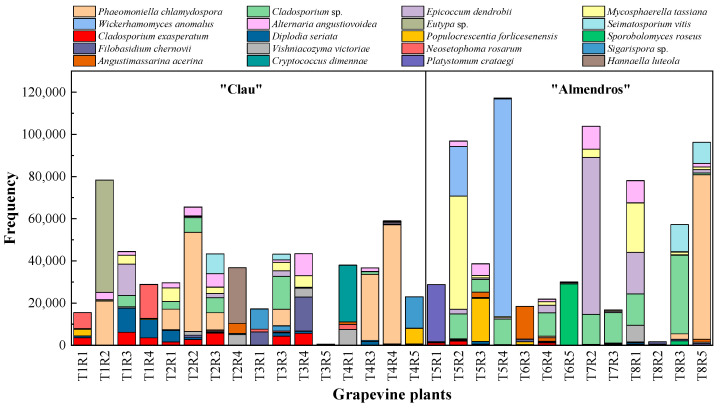
Stacked column histogram showing the twenty most frequent OTUs, ranked by abundance, in the two vineyards. The red line separates the plants from the two plots: (**left**) “Clau” vineyard, (**right**) “Almendros” vineyard.

As previously mentioned, *Phaemoniella chlamydospora*, a vascular pathogen associated with Petri disease, was the most abundant taxon in the entire metagenomic analysis; it was also the most abundant considering the two vineyards separately. Along with the aforementioned species, some of the most frequent OTUs in the analysis (Figure 4) were pathogens associated with the grapevine plant, including *Eutypa* sp., *Diplodia seriata*, and *Seimatosporium vitis*, the first two being specifically associated with GTDs. The analysis also identified two taxa commonly reported as microbial antagonists, namely the cosmopolitan endophyte *Epicoccum dendrobii* Q. Chen, Crous and L. Cai, and the yeast *Wickerhamomyces anomalus* (E.C. Hansen) Kurtzman, Robnett and Basehoar-Powers, among the 10 most frequent OTUs in the analysis, along with saprophytic or facultative parasitic taxa of the genus *Cladosporium*. *Mycosphaerella tassiana* (De Not.) Johanson, an ascomycete commonly reported as part of the fungal component of the microbial communities of the crops in numerous previous studies [11,31], which was also among the most frequent OTUs identified in the analysis.

**Figure 5:**
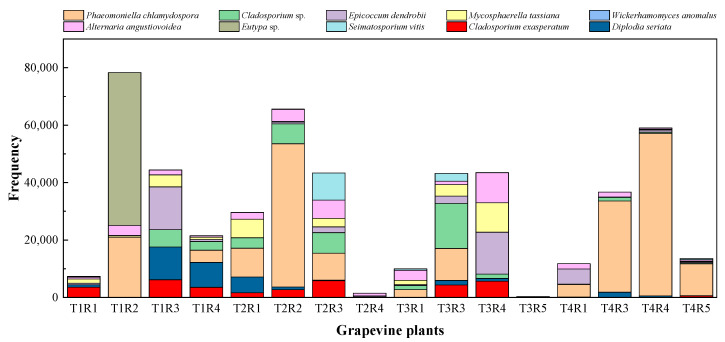
Stacked column histogram showing the ten most frequent OTUs, ranked by abundance, identified in the “Clau” vineyard.

**Figure 6:**
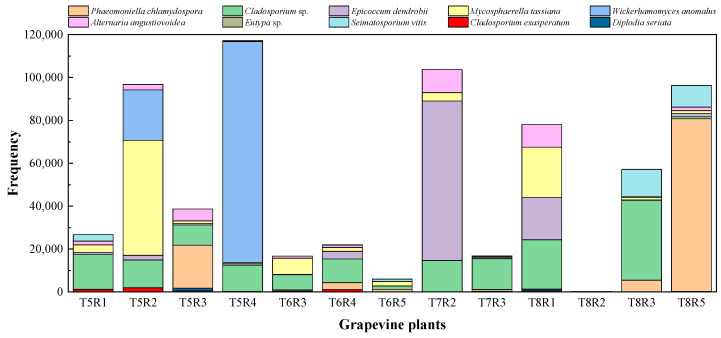
Stacked column histogram showing the ten most frequent OTUs, ranked by abundance, identified in the “Almendros” vineyard.

When the ranking of the most frequent OTUs was analyzed in each plot separately, notable differences were observed in their distribution and abundance according to the age of the vineyard. The distribution of high frequencies of appearance was quite irregular. *Phaeomoniella chlamydospora*, the most abundant taxon in the study, was uniformly distributed in all the plants sampled from the oldest plot (“Clau”) (Figure 5), being present in most of the plants analyzed, although with higher frequency in individuals belonging to row 4. However, *P. chlamydospora* was only detected in five plants of the youngest vineyard (“Almendros”) (Figure 6), with generally low values, except for the case of a specific plant in row 8. *Eutypa*, another pathogen associated with GTDs, only appeared in two individuals, one in each vineyard. However, it appeared with different frequencies, being very abundant in a plant from row 1 of the oldest plot and appearing in an almost testimonial way in a plant from row 7 in the youngest vineyard. *Diplodia seriata*, one of the etiological agents related to ‘Botryosphaeria dieback’, was more abundant and homogeneously represented in the older plot, appearing in almost all the plants sampled from the “Clau” vineyard, while it was detected in the younger vineyard only in five plants, with low values of appearance frequency.

As expected, the most frequent species representing common and cosmopolitan endophytic taxa such as *Epicoccum dendrobii*, *Cladosporium* spp., or *Alternaria angustiovoidea* E.G. Simmons were present in almost all the plants analyzed with different frequency rates, regardless of the location and age of the plots.

**Figure 7:**
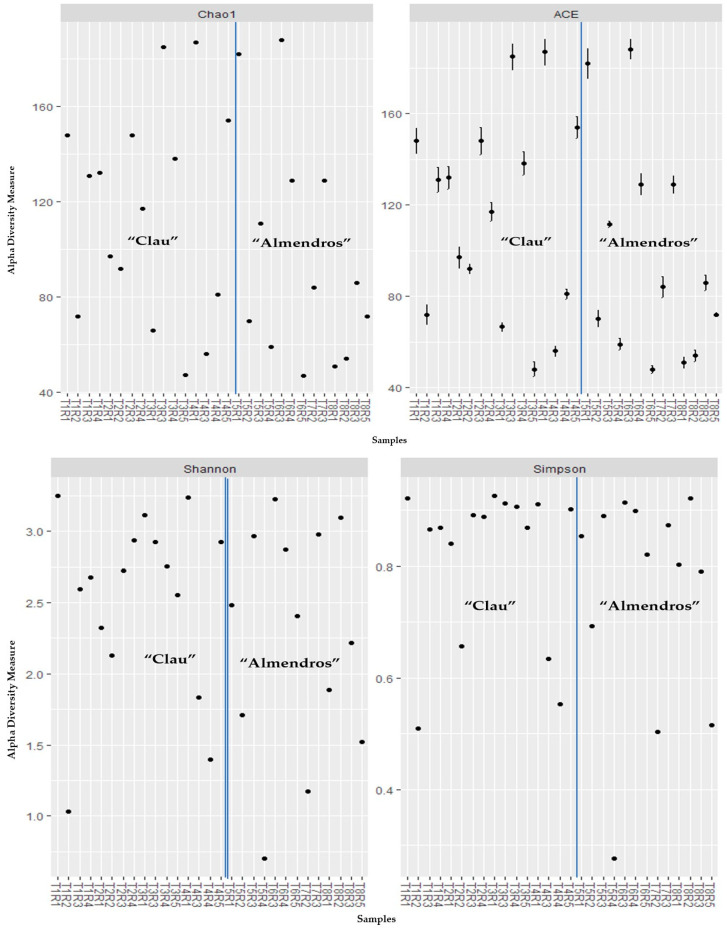
Alpha diversity and richness indexes for the fungal communities present in each sample: (**top**) Chao1 and ACE; (**bottom**) Shannon and Simpson. Blue lines separate plants from the two plots analyzed; T1–4 come from the “Clau” vineyard; T5–8 come from the “Almendros” vineyard.

An alpha-diversity analysis did not reveal significant differences in diversity and richness indexes (Figure 7) at the OTU level between the fungal communities of both vineyards. The Chao1 and ACE indices, which reflect the abundance of OTUs in the different samples (plants analyzed), had high ranges of values (48 to 175) for both vineyards, indicating a varying richness of species in each individual analyzed within the same plot or even the same row. The Shannon and Simpson indexes, which reflect the diversity of the OTUs in the samples, indicated that the fungal microbial communities associated with the inner grapevine wood in both vineyards have normal to low diversity values. The majority of values for the Shannon index (H) ranged between two and three, with only a few plants per plot presenting H values greater than three. For the Simpson index, in which higher values denote a lower diversity of the microbiome associated with each sample, most of the plants sampled in both plots had values between 0.8 and 1.0, suggesting discrete rates of microbial diversity.

#### 2.2.2. Comparison between Vineyards: Beta-Diversity

**Figure 8:**
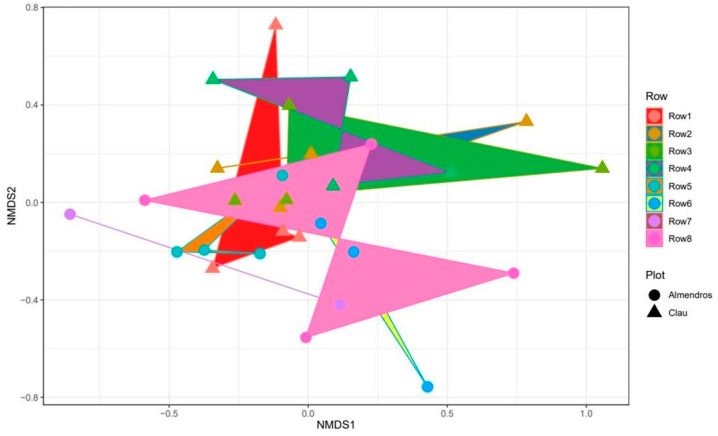
Non-metric multidimensional scaling (NMDS) plot of the metagenome of wood samples from the two surveyed vineyards. Samples in the different groups are represented by the symbols given in the legend, and colors represent the rows in the vineyard plots. Rows 1–4 come from the “Clau” vineyard; rows 5–8 come from the “Almendros” vineyard.

The difference in the composition of fungal communities between the analyzed vineyards was reflected in the beta-diversity analysis. Thus, a non-metric multidimensional scaling (NMDS) plot (Figure 8) revealed that the fungal microbial communities of the two vineyards sampled were not essentially different when considering each row of plants analyzed per plot, where an overlap was observed between the different groups. However, the communities associated with most of the rows and plants of the youngest vineyard (“Almendros” plot) exhibited a certain degree of differentiation from the rest of the samples analyzed, including all the rows sampled in the “Clau” plot, the oldest one.

**Figure 9:**
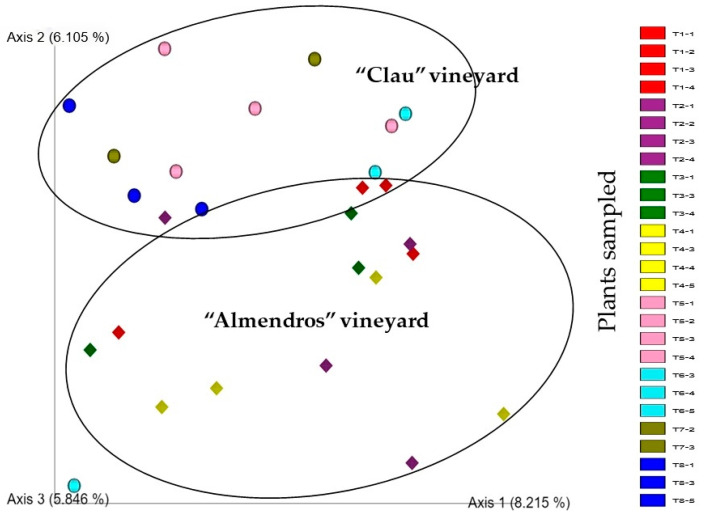
Emperor PCoA plot using Jaccard’s index of the metagenomes of the wood samples analyzed. The T1–4 (spheres) are samples from the “Clau” vineyard; the T5–8 (diamonds) are samples from the “Almendros” vineyard. Different colors represent different rows.

When a principal coordinate analysis was carried out to assess beta-diversity based on the Jaccard’s index, the resulting PCoA plot (Figure 9) revealed some dissimilarity between the samples from both vineyards. The plot showed that 8.21% of the data variability was explained by axis 1, reasonably separating the different communities associated with the sampled plants into two distinct groups with overlap, suggesting a differential fungal microbiome composition in grapevine inner wood tissues depending on the plot analyzed.

**Figure 10:**
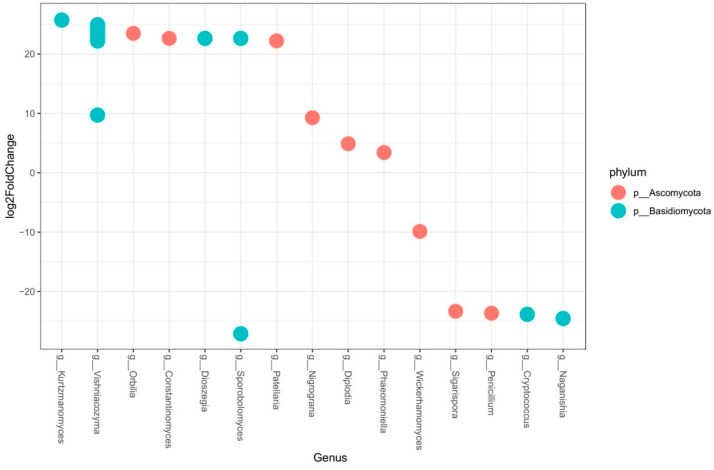
Pairwise comparison (DeSeq analysis). Differential abundance of OTUs (*p* < 0.05) in wood samples from the “Clau” and “Almendros” vineyards. OTUs were assigned to the genus level (*x*-axis) and phylum level (colors). For OTUs not defined at the genus level, the most specific taxonomic level available was used. Positive values indicate a higher abundance in the “Clau” vineyard and negative values indicate a greater abundance in the “Almendros” vineyard.

A detailed out-level base pairwise comparison between the “Clau” and “Almendros” vineyards was conducted, and it revealed 21 differences in the abundance of OTUs (Figure 10), with 15 in the “Clau” vineyard and 6 in the “Almendros” plot. The analysis identified several genera belonging to the phylum Ascomycota, such as *Orbilia*, *Constantinomyces*, *Patellaria*, and *Nigrograna*, as well as GTD pathogens *Diplodia* or *Phaeomoniella*, and genera of the phylum Basidiomycota, such as *Kurtzmanomyces*, *Vishniacozyma,* or *Dioszegia* that were dominant in the oldest plot. In contrast, the genera of ascomycetes such as *Wickerhamomyces*, *Sigarispora*, or *Penicillum*, and basidiomycetes such as *Cryptococcus* or *Naganishia* were enriched in the samples of the youngest vineyard. This type of analysis indicated that some genera related to GTDs such as *Diplodia* and *Phaeomoniella*, which are associated with Botryosphaeria dieback and Petri disease, respectively, were more abundant in the older plot, along with a series of basidiomycete yeast-like genera such as *Kurtzmanomyces*, *Dioszegia*, or *Vishniacozyma* (the most abundant yeast associated with the mentioned vineyard). On the other hand, yeast-like genera such as *Cryptococcus* and *Naganishia*, both basidiomycetes, or *Wickerhamomyces*, an ascomycete, were more abundant in the younger plot.

### 2.3. Correlation between NGS and Culture-Dependent Methods

During the analysis of taxonomic data obtained through culture-dependent microbiological methods in the same grapevine plants that were later analyzed by massive sequencing techniques, a low correlation rate was observed between the most frequent taxa that comprised the entire fungal microbiome of the woody tissues of the plants analyzed in both vineyards and the lists of endophytic species isolated and characterized using classical microbiological techniques. This suggests an important bias in the results obtained through classical microbiological techniques, where the microbial diversity revealed appears to be much lower and limited compared to the metagenomic results. Many endophytic taxa isolated and identified from both the “Clau” vineyard (Table S1) and the “Almendros” plot (Table S2) were part of the lists of OTUs obtained by high-throughput sequencing in the two plots, except for species such as *Rhizoctonia solani* J.G. Kühn, *Discostroma fuscellum, Didymella glomerata, Didymosphaeria variabile, Phomopsis viticola,* or *Phoma conidiogena*. However, the frequencies of appearance of the taxa common to the two approaches were found to be comparable only in certain cases. Among the ranked list of the 10 most frequent OTUs in the “Clau” vineyard (Figure 5), only the GTD pathogen *Diplodia seriata* was found to be frequent in the culture-dependent analyses (representing approximately 33% of the isolates from the plot), together with moderately frequent taxa such as *Epicoccum* sp. and *Eutypa lata* (representing approximately 9% and 4.5% of the isolates, respectively, in the microbiological survey). Despite being the most abundant taxon in the metagenomic analysis, the pathogenic species *Phaeomoniella chlamydospora* (representing 4.5% of the total number of isolates) was scarcely represented in the list of isolates from the older plot. Another surprising result was that *Neofusicoccum parvum*, one of the species frequently associated with GTDs and isolated in axenic cultivation of the “Clau” vineyard (representing 20% of the total isolates), was not among the most frequent OTUs defining the microbiome of the aforementioned plot. A similar situation was observed for another species associated with the early stages of grapevine esca, *Phaeoacremonium aleophilum*, which represented 4.4% of the total isolates but was not among the most frequent OTUs in the inner wood microbiome of the plants analyzed in the oldest vineyard.

When this type of comparison was established in the youngest vineyard (“Almendros” plot), the data turned out to be similar, although the percentage of strains representing the taxa isolated in cultivation common to the ranking of the 10 most frequent OTUs in the microbiome of the “Almendros” vineyard was, in general, lower than for the older plot, suggesting a replacement of the dominant strains in the microbiological analysis. Thus, *Epicoccum* sp. represented 25.7% of the total isolates, followed by *D. seriata* (11.5% of the total), together with *N. parvum* and *E. lata* with percentages of the total isolates of 5.7% and 2.8%, respectively. Interestingly, *P. chlamydospora* was not isolated by microbiological methods from the youngest plot. As expected, another phytopathogen associated with GTDs in young grapevine plants such as *Cylindrocarpon* spp. (etiological agent of the so-called ‘black foot’ disease), although present in 4.4% of the isolates obtained in the “Almendros” plot (and absent from the oldest vineyard), did not appear as part of the microbiome of this last vineyard.

## 3. Discussion

### 3.1. Grapevine Inner Wood Microbiome

The grapevine crop is one of the many agroecosystems that has been analyzed for its associated microbial diversity in the past decade using high-throughput sequencing techniques. While these analyses have primarily focused on the microbial communities inhabiting above-ground plant tissues [26,32,33,34], there has been some examination of the microbial diversity present in both the rootstock and root tissues [35,36]. In the production area under study, as well as in all Spanish viticultural areas, GTDs pose a serious and growing problem that threatens the profitability of farms [1]. With new pathologies emerging [37] and previously known syndromes persisting [38], there is a risk to both established plantations and new ones. The implementation of more precise and extensive diagnostic and epidemiology techniques has allowed for the characterization of the global panorama of GTDs in recent years, identifying entry routes, management alternatives, control possibilities, and other factors of these pathologies [39]. In terms of NGS techniques, these have primarily contributed to broadening our knowledge of the microbial diversity associated with the grapevine plant, greatly increasing the taxonomic inventories of microorganisms (fungi and bacteria) that can be found living inside the various tissues, organs, and compartments of the vine. NGS techniques have also aided in clarifying and resolving key questions related to the etiology and population dynamics of the etiological agents involved in GTDs, including the relationships between pathogen populations and the expression of foliar symptoms in plants [26], the underestimation of the pathogenic species present in a certain vineyard, and the precise detection of latent infections in crops [40].

The metagenomic analysis of the vineyards considered in this study assigned a total number of 1300 OTUs to 318 different fungal species. The number of characterized taxa in our study falls within the range of those obtained in previous works that focused on the microbiome of different organs of the grapevine plant. For instance, in the study by Wei et al. [21], 569 species of fungi were characterized on leaves, 376 in the case of rootstock-associated fungal diversity in the work by Gramaje et al. [35], 289 in the study carried out by Del Frari et al. [26] on the internal wood microbiome of plants affected by esca, and 732 OTUs were detected in the study by Lade et al. [41], where the fungal communities associated with graft unions and root collars were characterized in propagation plant material from nurseries. As expected, the fungal microbiome in the two plots considered in this study was dominated by Ascomycota, while Basidiomycota represented a minor fraction (less than half of the above), as previously reported in numerous studies on grapevine employing similar approaches [26,32,42]. It is important to highlight that, although the plants analyzed were chosen based on the presence of foliar symptoms, decay, and wood rot, the metagenomic analyses did not detect any of the lignicolous basidiomycete taxa that are traditionally associated with the advanced stages of complex syndromes such as esca, even in the oldest vineyard (23 years old). However, this finding aligns with the results of Bekris et al. [9] who found an increase in the distribution and abundance of this type of taxa (mainly *Fomitiporia mediterranea*) in the symptomatic plants they analyzed. Paolinelli et al. [43] and Del Frari et al. [44] also observed this increase in the presence of lignicolous basidiomycetes in symptomatic plants, suggesting that an increase in this type of species can serve as an early indicator of the extension of this type of GTD. Regarding other taxa associated with GTDs, numerous species were identified, with some being extremely frequent (as in the case of *Phaeomoniella chlamydospora*) and others being less represented, such as species associated with the so-called Petri disease, including *Phaeoacremonium aleophilum*, *P. minimum,* or *Cadophora luteo-olivacea*; etiological agents associated with ‘Botryosphaeria dieback’ (including representatives of the genera *Neofusicoccum*, *Dothiorella*, or *Diplodia*); or the causal agent of eutypiose (*Eutypa lata*). This finding is consistent with the results of authors such as Del Frari et al. [26] who found a similar profile of ascomycete species associated with the internal wood of the vine.

In our study, approximately 80% of the characterized taxa turned out to be rare species, with a relative abundance of less than 2%. This is similar to what was observed by Del Frari et al. [26] who reported that in their analysis of the internal wood microbiome of grapevine plants affected by esca, 80% of the characterized taxa exhibited a relative abundance of less than 0.1%. In both our study and that of Del Frari et al., many of these rare or occasional species were well characterized from the point of view of their ecology, but an important contingent of them is either not specifically associated with the host or its role in the plant has yet to be elucidated. This observation suggests the hypothesis of the existence of a reservoir of rare and occasional diversity that, under the presence of certain drivers such as the triggering of specific environmental conditions (both biotic and abiotic), can play a more relevant role in the well-being and ecological fitness of the plant. This hypothesis aligns with the principles and functioning of the holobiont concept [6].

Many metagenomic studies on grapevines have aimed to identify “core” microbial populations driven by factors such as biotic or abiotic stress [45], plant genotype [46], or crop age [18]. In this study, we identified a core fungal community shared between the two vineyards analyzed, consisting mostly of pathogenic taxa and other generalist or ubiquitous endophytic species, as well as several OTUs associated with the plant host. In this sense, and when considering the phytosanitary status or age of the hosts analyzed, some studies were equally capable of characterizing essential and common microbial communities in plants of different geographical locations and ages. For example, Berlanas et al. [18] reported the existence of a core population that was common to the rootstocks of two vineyards geographically separated and of very different ages, 25 and 7 years old, respectively, with planting ages very similar to those of our case study. Similarly, Del Frari et al. [26] reported in a study on the microbiome associated with inner wood and canes of grapevine plants affected by esca that the fungal core community common to both types of woody tissues was dominated, as in the present study, by the same spectrum of pathogenic taxa associated with GTDs, together with a series of cosmopolitan and ubiquitous endophytic OTUs. Among this core community, the study highlights, as in our metagenomic analysis, the presence of *Phaeomoniella chlamydospora* as the most abundant component in this essential population. Likewise, Niem et al. [22] reported in a metagenomic study on symptomatic and asymptomatic grapevine plants in two vineyards in Australia that *Phaeomoniella chlamydospora* was the most abundant species, being even more frequent in asymptomatic plants. These authors found, as in our analysis, that the microbiome was dominated to a lesser extent by *Botryosphaeriaceae* species, certain lignicolous basidiomycetes, cosmopolitan endophytes, or species with potential as BCAs. Bekris et al. [9], in a study on the wood microbiome of grapevines from three Greek cultivars located in three geographically distinct viticultural zones, reported that both geographical location and plant genotype were the determinants of fungal diversity composition, not phytosanitary status, unlike what is reported in other studies. Despite this, these authors also identified *P. chlamydospora* as one of the main agents responsible for the GTDs present in the study. Finally, Patanita et al. [31], in a metagenomic approach to fungal microbial communities associated with healthy grapevine plants and common symptoms of GTDs, found that taxa such as *Diplodia* sp., *Mycosphaerella tassiana*, *Alternaria* sp., or *Cladosporium* sp. were the most abundant in the study, similar to what was reported in our analysis.

### 3.2. Comparison of Microbial Communities: Beta-Diversity

A key aspect of this study is comparing the microbial diversity associated with two vineyards of different ages and locations with a high prevalence of plants with symptoms compatible with the presence of GTDs. Our results suggest that the age of the plantation is the main factor that could explain the differences detected between both microbiomes. In general terms, the fungal microbiome in the older plot was found to be more diverse and complex than in the eight-year-old vineyard. Thus, a differential composition of OTUs was found in both communities, where some GTD-genera such as *Diplodia* and *Phaeomoniella* were more abundant in the older plot, along with a series of basidiomycete yeast-like genera such as *Kurtzmanomyces*, *Dioszegia*, or *Vishniacozyma*. On the other hand, yeast-like genera such as *Cryptococcus* and *Naganishia*, both basidiomycetes, or *Wickerhamomyces*, an ascomycete, were more abundant in the younger plot, as well as certain taxa associated with GTDs mainly found in young plants, such as those related to the so-called ‘black foot’ disease. Several studies, such as the one by Dissanayake et al. [47], have revealed a positive correlation between grapevine age and fungal endophytic diversity, suggesting an increase in the diversity and complexity of these populations with an increase in the exposure time to these microorganisms. In addition, Berlanas et al. [18], although focused on the fungal rhizosphere microbiome, also reported that the diversity of rhizospheric microorganisms could be affected by plant age, although this was not the most important factor when it came to differentially modeling the microbiome associated with the rootstocks of young and mature plants.

### 3.3. Comparative Microbial Diversity According to Methodology: NGS vs. Culture-Dependent Techniques

The present study compared the diversity of endophytic fungi (including those that are GTD pathogens) associated with the interior of vine plant wood through culture-dependent methods and NGS techniques. The results showed that the diversity of cultivable species inside the plant was significantly lower than that characterized by metagenomics, as expected and as shown in numerous previous studies [47,48]. Furthermore, no clear correlation was found between the composition of isolated endophyte communities in the pure culture and the most frequent OTUs characterized in the metagenomic analysis, suggesting a bias when using classical microbiological methods. These methods are only capable of characterizing the cultivable mycobiota, which represents a very minor portion of the total number of fungi associated with the host plant. Despite this, some taxa (especially those with pathogenic behavior) isolated by microbiological methods had a comparable abundance between methods since they were part of the most frequent OTU rankings in the NGS analysis. In this sense, in an analysis of the fungal diversity of the aerial parts of grapevine plants of the ‘Furmint’ variety in Hungary, Knapp et al. [32] found a notable difference between the number of species resolved by both methods (being clearly higher in the metagenomic analysis). However, as in the present study, the core communities of microorganisms shared species in both taxonomic inventories. Similar results have been reported when employing other types of massive, indirect high-throughput analyses. In the work of Morales-Cruz et al. [40], the authors analyzed the microbial diversity of grapevine plants affected by GTD at various locations in California using the sequencing of ribosomal DNA transcripts (rRNA) in a metatranscriptomic approach. They also reported that metagenomic and metatranscriptomics approaches revealed much greater species complexity than that obtained by direct fungal isolations.

## 4. Materials and Methods

### 4.1. Grapevine Plots

**Figure 11:**
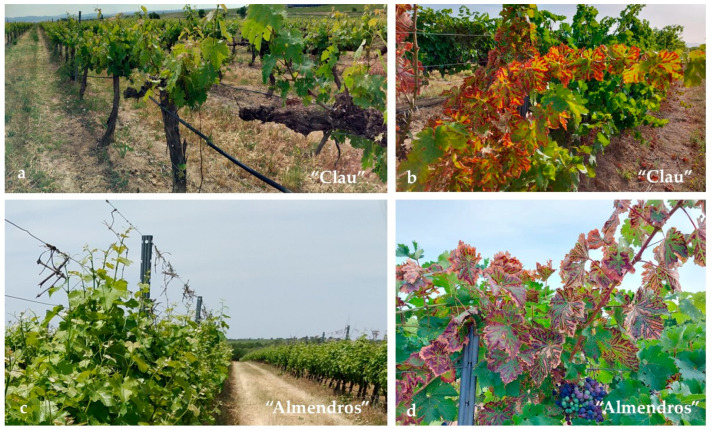
Commercial vineyards studied in this work. Photographs on the left show general views of the plots in May 2021 and photographs on the right show details of GTD foliar symptoms. (**a**,**b**): “Clau” vineyard; (**c**,**d**): “Almendros” vineyard.

The samplings were carried out in two commercial vineyards located in the Huesca province in Northeastern Spain. The vineyards, “Clau” (at 41°59′39.2″ N; 0°08′04.5″ E) and “Almendros” (at 41°59′01.7″ N; 0°07′33.9″ E) belong to the PDO “Somontano” and represent two plots that differ in their year of plantation. Both vineyards have phytosanitary problems associated with varied symptoms attributable to GTDs, such as interveinal “striping”, inner wood rotting, or entire branch collapse (Figure 11). Additional information on cultivar type, bioclimate parameters, management practices, and other relevant data can be found in Table 2.

### 4.2. Wood Samples

In each of the two vineyards studied, a total of 20 grapevine plants were sampled. Specifically, five plants per row were chosen for sampling, with four rows sampled in total. The chosen plants had previously been marked as diseased due to the presence of GTD symptoms, such as foliar chlorosis, stunted growth, and wood rot. To characterize the fungal microbiome of the inner wood, each marked plant was sampled at the point of separation of both arms. This was achieved by drilling a hole approximately 4 mm in diameter and 8 cm deep and collecting all the plant material extracted (approximately 2 g) into plastic envelopes while trying to discard the bark. Finally, the inner wood samples were refrigerated at 4–6 °C before being taken to the laboratory, where they were stored at −20 °C until subsequent DNA extraction.

### 4.3. Isolation of Grapevine Endophytic Fungi

Grapevine plants were sampled for their aerial tissues (vine shoots and arms) to isolate and characterize the different fungal species associated with them (including GTD-associated fungi) using microbiological culture-dependent methods. To do this, plant material (either shoot discs or inner wood blocks) was cut into 0.5 cm fragments and surface sterilized with a 70% EtOH solution (1 min), followed by a 5% commercial sodium hypochlorite solution (3 min), and then rinsed with sterile bi-distilled water 3–5 times. The fragments were then placed in PDA plates (CULTIMED, Barcelona, Spain) amended with streptomycin sulfate (0.3 g/L) to avoid bacterial contamination and incubated in the dark at 25 °C for 3–5 days to obtain emerging fungal colonies. The resulting colonies were transferred to new PDA plates to obtain pure cultures of each endophytic strain. Finally, these isolates were taxonomically identified by applying both morphometrical and molecular methods, including comparing their ribosomal ITS fragment sequences in public databases using the BLASTn tool [49].

### 4.4. DNA Extraction and Sequencing

Prior to performing total genomic DNA extraction, the wood samples were deep-frozen for 30 min at −85 °C, then crushed in a mortar and pestle with liquid nitrogen, and subsequently lyophilized for 72 h in a Cryodos-80 device (Telstar, Barcelona, Spain). Then, approximately 250 mg of dried powdered wood per plant sample was used to extract total genomic DNA with the DNeasy^®^ Plant Mini Kit (Qiagen, Germany), following the manufacturer’s instructions. The concentration of extracted DNA was measured using a NanoDrop^®^ ND-1000 spectrophotometer device (Whaltam, Thermo Scientific, MA, USA). The integrity of DNA was assessed through 1.5% agarose gel electrophoresis. Samples were then sent to Stab Vida, Lda. (Caparica, Portugal) for NGS analysis. The ITS (ITS2 fragment) region was selected as the target amplicon for the metagenomic study of the fungal community. Due to the existence of suboptimal total DNA concentrations in some samples, the genomic libraries and subsequent massive sequencing analyzes were performed on 16 of the 20 plants in the “Clau” plot and on 13 of the 20 samples from the “Almendros” plot. In both cases, all the sampled rows were analyzed in at least one of their repetitions. The Illumina metagenomic sequencing library preparation protocol was used to construct the library, and the resulting DNA fragments were sequenced on the Illumina MiSeq platform using 300 bp paired-end reads.

### 4.5. Bioinformatics Procedure

After sequencing, the generated raw data (6,498,688 reads total, 105,964 to 361,014 raw sequence reads per sample) were downloaded and processed through a galaxy workflow using DADA2 (Galaxy Version 1.20+galaxy0) [50]. The sequences were trimmed and quality filtered, the error rate was denoised, and bimeras were removed. The reads were then grouped into OTUs and classified by taxon using the DADA2 modules assignTaxonomy and addSpecies and the 8.3 version of the general fasta release of the UNITE database [51]. The processed OTU table was composed of 2,386,482 reads from 1300 OTUs.

### 4.6. Statistical Data Analysis

Statistical analysis was performed in Rstudio using the phyloseq package [52]. Alpha-diversity was determined using four diversity indexes (Chao, Shannon, Fisher, and Simpson). For beta-diversity analysis purposes, principal coordinate analysis (PCoA) and ordination plots of OTU data were constructed using the Phyloseq program for R. The DESeq2 package was used to detect differentially abundant OTU between rows and plots with default parameters (Wald test). To delve deeper into the effects of alternate mycobiota communities in the different plots, FUNGuild annotation tools were utilized for functional predictions [53]. The different Venn diagrams showing the intersections between OTUs belonging to different vineyards and rows within each plot were generated with the ‘Venn Diagrams’ tool [54].

## 5. Conclusions

High-throughput sequencing enabled the characterization of the fungal microbiota associated with the grapevine internal plant wood tissues in two experimental vineyards, selected based on their planting age, phytosanitary status, and cultivated variety. In addition, an analysis of the fungal diversity existing in the same woody tissues was conducted using culture-dependent microbiological techniques. The metagenomic analysis revealed a high fungal diversity compared to classical taxonomic methods in terms of the number of characterized OTUs, abundance, and frequency of occurrence. The taxonomic results from classical microbiological methods did not reflect the true diversity of the endophytes associated with internal plant wood or the composition and population structure of this microbial diversity. The study of the microbiome demonstrated that the symptoms associated with GTDs could be attributable to the dominant presence of *Phaeomoniella chlamydospora* and, to a lesser extent, *Diplodia seriata*. However, culture-dependent methods did not yield the same view in terms of the abundance of isolates belonging to both taxa. In general, the microbiota of the older plot was more diverse and complex, being dominated at the level of pathogenic taxa by those associated with wood pathologies characteristic of mature plants, while in the young vineyard, elements associated with GTDs that preferentially affect young plants dominated. The results suggest a differential replacement and enrichment of fungal microbial communities based on age and exposure to endophytic infection, where the dominant species in each type of microbiota could have been prioritized by plant individuals based on the age, anatomy, and structure of their tissues. In general terms, the results of the metagenomic analysis revealed a characteristic mycobiota of the internal wood (and, to a lesser extent, of other plant organs) consisting of microorganisms repeatedly cited in previous studies of a similar nature, suggesting a priming effect promoted by the grapevine plant that is modulable according to each genotype in question, management modality, or the age of the crop.

## Figures and Tables

**Table 1:** Fungal OTUs common to all grapevine plant rows analyzed (including both vineyards). Taxa followed by a “V” in parentheses indicate those specifically associated with *Vitis vinifera* L. Data on the relative frequency and fungal guild are provided.

Taxonomy	Frequency	Fungal Guild
*Cladosporium allicinum*	4601.6	Pathotroph
*Epicoccum nigrum* (V)	5101	Pathotroph–Saprotroph–Symbiotroph
*Stemphyliuym majusculum*	148.1	Pathotroph–Saprotroph
*Cystofilobasidium macerans* (V)	138	Saprotroph
*Seimatosporium vitis* (V)	1414.3	Pathotroph
*Phaeomoniella chlamydospora* (V)	12,741.1	Pathotroph
*Filobasidium stepposum* (V)	1028.8	Saprotroph
*Knufia perforans*	308.9	Pathotroph–Saprotroph
*Aureobasidium pullulans* (V)	683	Pathotroph–Saprotroph–Symbiotroph
*Filobasidium magnum* (V)	69.2	Saprotroph
*Rhinocladiella* sp. (V)	175	Pathotroph
*Cladosporium grevilleae* (V)	358.1	Saprotroph
*Cyphellophora* sp.	174.9	Pathotroph–Saprotroph
*Cladosporium cladosporioides* (V)	8257.4	Saprotroph
*Angustimassarina acerina* (V)	954.7	Saprotroph
*Myrmecridium banksiae*	65.3	Saprotroph
*Vishniacozyma victoriae* (V)	122.4	Saprotroph
*Aspergillus undulatus*	643.2	Pathotroph–Saprotroph
*Populocrescentia forlicesenensis*	1198.2	Pathotroph–Saprotroph
Ord. *Malasseziales*	23.2	Pathotroph–Saprotroph
*Acericola italica*	410.6	Pathotroph–Saprotroph
*Sporobolomyces roseus*	128.8	Pathotroph–Saprotroph
*Neoscytalidium* sp. (V)	43.8	Pathotroph–Saprotroph
*Alternaria infectoria*	359.2	Pathotroph–Saprotroph–Symbiotroph
*Neosetophoma lunariae* (V)	289.2	Pathotroph
*Vishniacozyma carnescens* (V)	751.5	Saprotroph
*Cladosporium exasperatum*	1392.3	Saprotroph
*Alternaria alternata* (V)	2608.3	Pathotroph–Saprotroph–Symbiotroph
*Cyphellophora oxyspora*	14.4	Pathotroph–Saprotroph
*Knufia mediterranea*	221.7	Pathotroph–Saprotroph

**Table 2:** Soil, bioclimatic, and agronomic data of the two vineyards surveyed.

Plot Name	“Clau”	“Almendros”
Var./Rootstock	“Cabernet Sauvignon” clone 170/SO4	“Sauvignon Blanc”/376 and R140
Year Established	2000	2015
Management/ Plantation frame	Cover crop, trellis formation system, and double cordon/3 × 1 m	Cover crop, trellis formation system, and double cordon/3 × 1.2 m
Soil Type	Loam texture; calcisol (accumulation of calcium carbonate at a certain depth, basic pH)	Loam texture; calcisol
Height (m.a.s.l)	375	401
Temperature, Rainfall, and Climate	14.23 °C, 486 mm, and continental Mediterranean climate	13.00 °C, 486 mm, and continental Mediterranean climate
Average Yield (last 3 years)	6000 kg/ha	9000 kg/ha

## Data Availability

The data presented in this study are available on request from the corresponding author. The data are not publicly available due to their relevance to an ongoing Ph.D. Thesis.

## Supplementary Materials

The following supporting information can be downloaded at: https://www.mdpi.com/article/10.3390/plants12122251/s1, Table S1: Fungal OTUs isolated by microbiological methods and identified from surveyed plants in the “Clau” vineyard; Table S2: Fungal OTUs isolated by microbiological methods and identified from surveyed plants in the “Almendros” vineyard; Table S3: Taxonomy table generated from NGS analyses. Total OTUs resolved indicating the appearance frequency per plant stand; Table S4: Ecology traits of the different OTUs characterized. For each taxon, indications about their trophic mode, guild, lifestyle, or growth form are given.
