# Supplementary material for: Metagenomic Study of Fungal Microbial Communities in Two PDO Somontano Vineyards (Huesca, Spain): Effects of Age, Plant Genotype, and Initial Phytosanitary Status on the Priming and Selection of their Associated Microorganisms
Source: Plants (Basel). 2023 Jun 8;12(12):2251. doi: 10.3390/plants12122251 (PMC10304032; doi:10.3390/plants12122251)
Supplement: Supplementary file 1 [file plants-12-02251-s001.zip › Tables S1 and S2.pdf]

Metagenomic Study of Fungal Microbial Communities in Vineyards of PDO Somontano (Huesca, Spain): Effects of Microbial Priming on Phytosanitary Traits of Different Grapevine Genotypes

Natalia Langa-Lomba, Jerome Grimplet, Eva Sánchez-Hernández, Pablo Martín-Ramos,
José Casanova-Gascón, Carmen Julián-Lagunas, and Vicente González-García

SUPPLEMENTARY MATERIAL

Table S1. Fungal OTUs isolated by microbiological methods and identified from surveyed plants in "Clau" vineyard.

ROW/PLANT	VINEYARD	ISOLATE
T 1,1	"Clau"	*Diplodia seriata*
T 1,1	"Clau"	*Neofusicoccum parvum*
T 1,1	"Clau"	*Diplodia seriata*
T 1,2	"Clau"	*Rhizoctonia solani*
T 1,2	"Clau"	*Neofusicoccum parvum*
T 1,2	"Clau"	*Diplodia seriata*
T 1,3	"Clau"	*Trichoderma ghanense*
T 1,4	"Clau"	*Eutypa lata*
T 1,4	"Clau"	*Diplodia seriata*
T 1,5	"Clau"	*Discostroma fuscellum*
T 2,1	"Clau"	*Neofusicoccum parvum*
T 2,1	"Clau"	*Diplodia seriata*
T 2,2	"Clau"	*Diplodia seriata*
T 2,3	"Clau"	*Trichoderma aureoviride*
T 2,3	"Clau"	*Gibberella fujikuroi*
T 2,4	"Clau"	*Diplodia seriata*
T 2,4	"Clau"	*Aureobasidium pullulans*
T 2,4	"Clau"	*Neofusicoccum parvum*
T 2,4	"Clau"	*Rhizopus* sp.
T 2,5	"Clau"	*Diplodia seriata*
T 2,5	"Clau"	*Epicoccum purpurascens*
T 2,5	"Clau"	*Neofusicoccum parvum*
T 2,5	"Clau"	*Didymella glomerata*
T 3,1	"Clau"	*Epicoccum purpurascens*
T 3,1	"Clau"	*Neofusicoccum parvum*
T 3,1	"Clau"	*Diplodia seriata*
T 3,2	"Clau"	*Phaeomoniella chlamydospora*
T 3,2	"Clau"	*Epicoccum purpurascens*
T 3,3	"Clau"	*Neofusicoccum parvum*
T 3,3	"Clau"	*Fusarium oxysporum*
T 3,3	"Clau"	*Phaeoacremonium aleophilum*
T 3,3	"Clau"	*Diplodia seriata*
T 3,4	"Clau"	*Diplodia seriata*
T 3,5	"Clau"	*Neofusicoccum parvum*
T 3,5	"Clau"	*Phaeoacremonium aleophilum*
T 3,5	"Clau"	*Diplodia seriata*
T 4,1	"Clau"	*Diplodia seriata*
T 4,2	"Clau"	*Neofusicoccum parvum*
T 4,2	"Clau"	*Gibberella* sp.
T 4,2	"Clau"	*Epicoccum purpurascens*
T 4,3	"Clau"	*Eutypa lata*
T 4,3	"Clau"	*Fusarium oxysporum*
T 4,4	"Clau"	*Diplodia seriata*
T 4,4	"Clau"	*Phaeomoniella chlamydospora*
T 4,5	"Clau"	*Diplodia seriata*

Table S2. Fungal OTUs isolated by microbiological methods and identified from surveyed plants in "Almendros" vineyard

ROW/PLANT	VINEYARD	ISOLATE
T 5,1	"Almendros"	*Fusarium oxysporum*
T 5,1	"Almendros"	*Gibberella fujikuroi*
T 5,1	"Almendros"	*Didymella glomerata*
T 5,2	"Almendros"	*Fusarium oxysporum*
T 5,3	"Almendros"	*Epicoccum purpurascens*
T 5,3	"Almendros"	*Neofusicoccum parvum*
T 5,4	"Almendros"	*Epicoccum purpurascens*
T 5,4	"Almendros"	*Trichoderma ghanense*
T 6,1	"Almendros"	*Neofusicoccum parvum*
T 6,1	"Almendros"	*Diplodia seriata*
T 6,2	"Almendros"	*Epicoccum purpurascens*
T 6,3	"Almendros"	*Aspergillus niger*
T 6,3	"Almendros"	*Cylindrocarpon macrodydimum*
T 6,4	"Almendros"	*Epicoccum nigrum*
T 6,4	"Almendros"	*Cylindrocarpon* sp.
T 6,5	"Almendros"	*Diplodia seriata*
T 6,5	"Almendros"	*Epicoccum nigrum*
T 6,5	"Almendros"	*Didymosphaeria variabile*
T 7,1	"Almendros"	*Epicoccum purpurascens*
T 7,2	"Almendros"	*Fusarium oxysporum*
T 7,3	"Almendros"	*Phomopsis viticola*
T 7,3	"Almendros"	*Epicoccum nigrum*
T 7,4	"Almendros"	*Trichodema viride*
T 7,4	"Almendros"	*Aspergillus* sp.
T 7,5	"Almendros"	*Diplodia seriata*
T 7,5	"Almendros"	*Epicoccum purpurascens*
T 8,1	"Almendros"	*Aspergillus niger*
T 8,1	"Almendros"	*Diplodia seriata*
T 8,2	"Almendros"	*Eutypa lata*
T 8,3	"Almendros"	*Phoma conidiogena*
T 8,4	"Almendros"	*Cylindrocarpon macrodydimum*
T 8,4	"Almendros"	*Epicoccum purpurascens*
T 8,5	"Almendros"	*Diplodia seriata*
T 8,5	"Almendros"	*Epicoccum purpurascens*
T 8,5	"Almendros"	*Alternaria* sp.
